# A case report of small cell neuroendocrine carcinoma of the ovary and review of the literature

**DOI:** 10.3389/fimmu.2025.1569011

**Published:** 2025-05-22

**Authors:** Ying Zhao, Fuli Kang, Xiangshu Kong, Ning Wang

**Affiliations:** Department of Gynecology and Obstetrics, Second Affiliated Hospital of Dalian Medical University, Dalian, China

**Keywords:** small cell neuroendocrine carcinoma, chemotherapy, immunotherapy, targeted therapy, malignant tumor of ovary

## Abstract

Small cell neuroendocrine carcinoma of the ovary is rare pathological type with an undefined mechanism, low incidence, but high metastatic rate, high aggressiveness, and very poor prognosis, and there are no standardized treatment protocols or guidelines. In this article, we report a 48-year-old woman diagnosed with small cell neuroendocrine carcinoma of the ovary after puncture biopsy of a pelvic mass, who underwent 8 cycles of paclitaxel+carboplatin regimen chemotherapy (with the addition of the anti-angiogenesis targeted agent bevacizumab in the first 2 times, and the immunosuppressant tirilizumab in the last 2 times), followed by 12 times of tirilizumab monotherapy maintenance therapy, which was highly effective. It is believed that with newer technologies, the use of surgery, chemotherapy combined with immunotherapy, targeted therapy, genetic testing and construction of animal tumor models will play a key role in the treatment and monitoring of the prognosis of this rare disease.

## Introduction

Neuroendocrine tumors account for <1% of all malignant tumors and are prevalent in the gastrointestinal tract, lungs, and a small proportion occur in the female reproductive system, among others (1). WHO classifies them into two categories: low-grade and high-grade. High-grade neuroendocrine tumors are further classified into small cell neuroendocrine carcinoma (SCNEC) and large cell neuroendocrine carcinoma (LCNEC).SCNEC accounts for only 2% of gynecologic malignancies and usually occurs in the uterine cervix and ovaries (2). Small cell neuroendocrine carcinoma of the ovary can be divided into 2 types: small cell carcinoma with hypercalcemia (SCCO with hypercalcemia type, SCCOHT) and small cell carcinoma of the lung (2). The incidence of ovarian small cell carcinoma is relatively low, but it progresses rapidly, with high malignancy, metastasis, recurrence and lethality. There is no specificity in the clinical presentation, and pathologic examination and immunohistochemical staining are required to confirm the diagnosis. Treatment options usually include surgery, platinum-based chemotherapy and radiotherapy, and the efficacy of targeted therapy and immunotherapy needs to be further studied. However, due to the rarity of the disease, there is no standardized treatment protocol. We report a case about chemotherapy combined with targeted and immunotherapy for small cell neuroendocrine carcinoma of the ovary, with significant therapeutic effects. Meanwhile, by searching PubMed, GeenMedical and other databases, we retrospectively analyzed the relevant studies (mechanism of occurrence, clinical manifestations, tumor markers, immunotherapy, and targeted therapy) of ovarian small cell neuroendocrine carcinoma in the last 20 years with a view to improving the prognosis of patients.

## Case description

**Figure 1:**
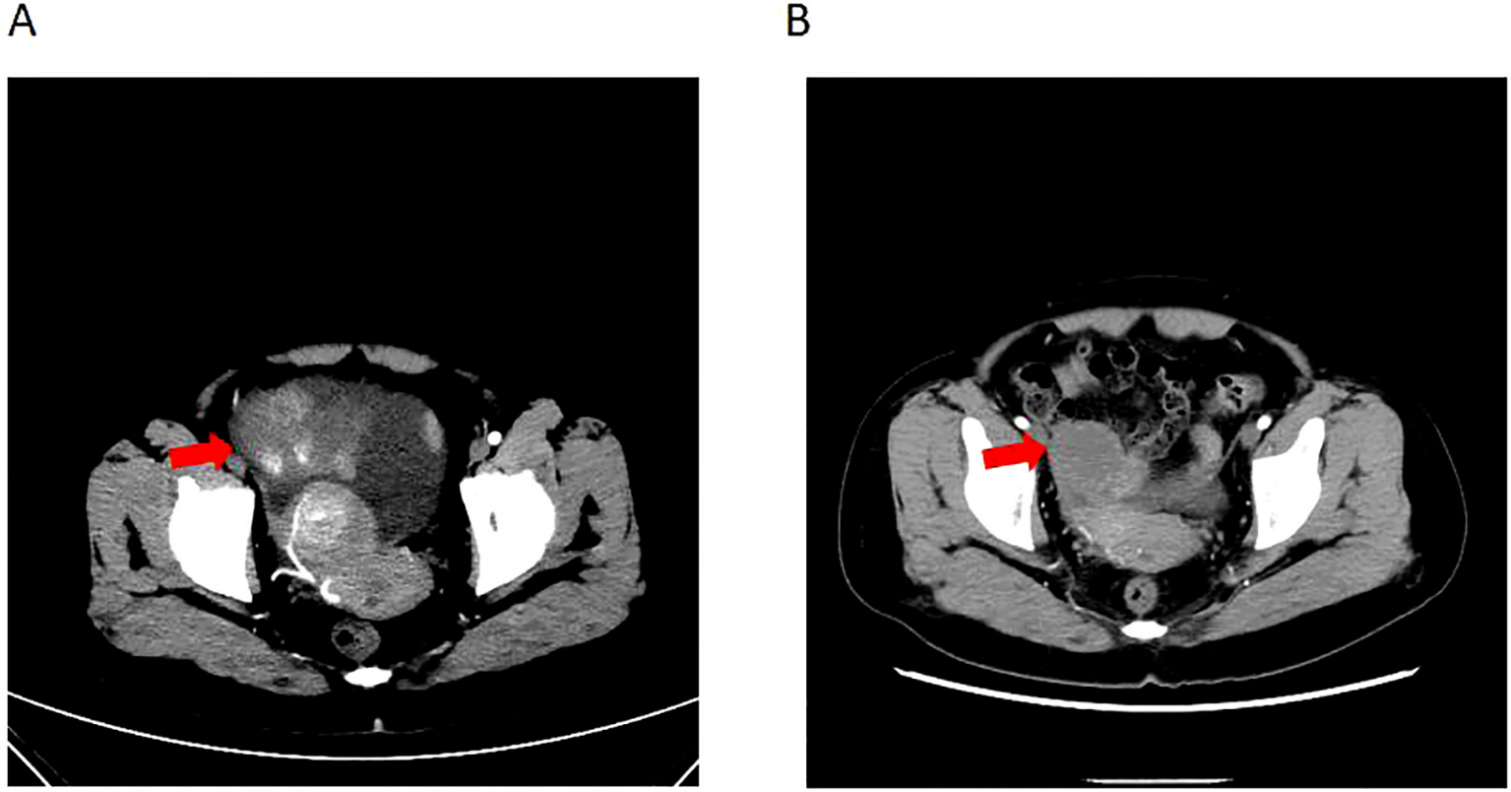
Computed tomography of ovary tumor before and after therapy. **(A)** CT of the irregular mass with dimensions of 8.3*6.5*5.5 cm and a large amount of fluid in the abdominal cavity and pelvis before therapy. **(B)** CT of irregular range is obviously regressed compared to before.

**Figure 2:**
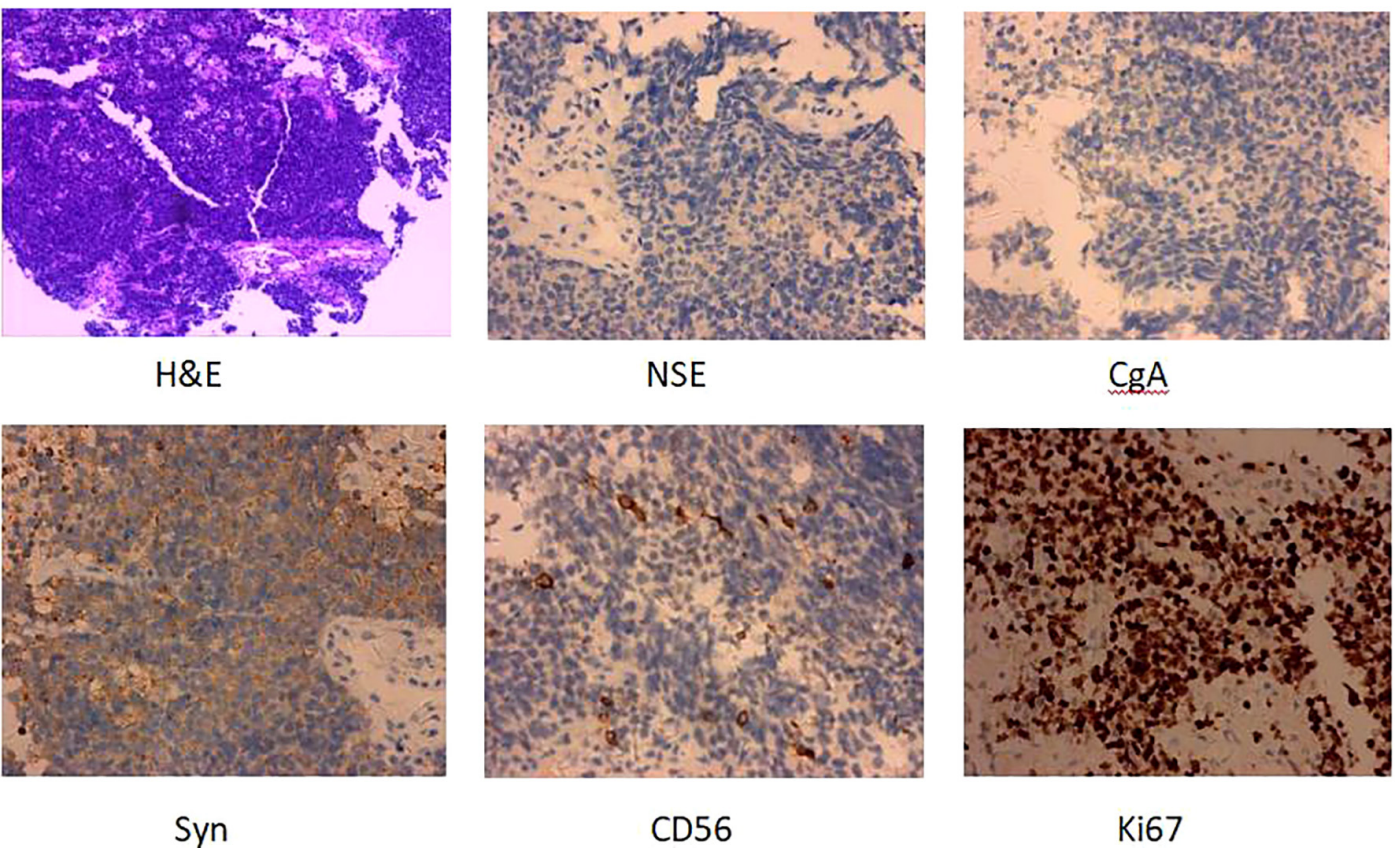
H&E and IHC of the patients’s pelvic mass puncture biopsy (H&E staining, ×100; IHC, ×100). H&E, Hematoxylin and eosin stain; IHC, immunohistochemistry.

**Figure 3:**
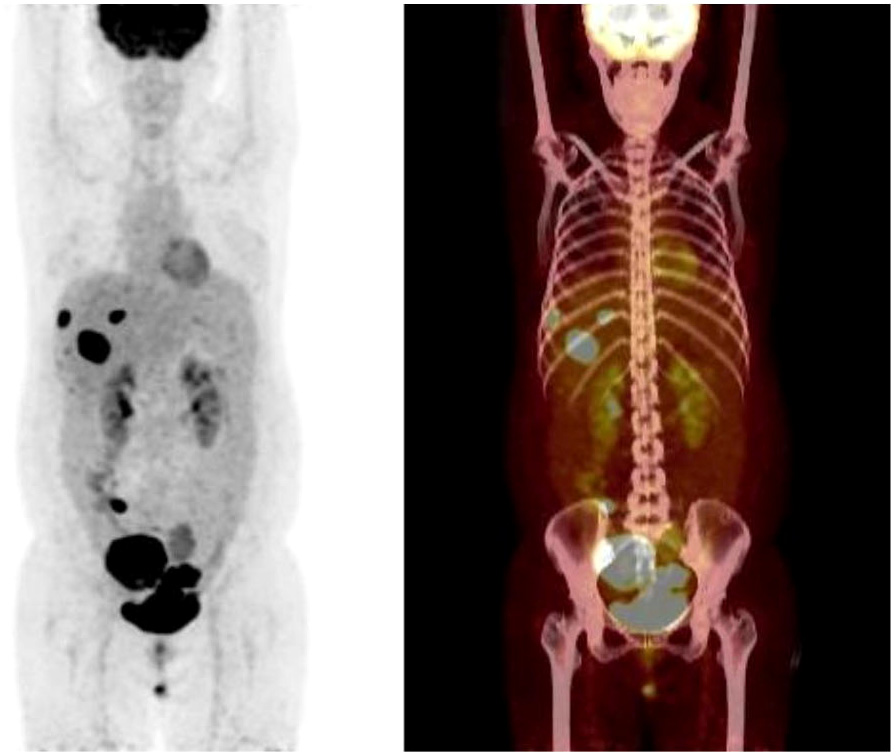
PET-CT shows: multiple metastatic lesions in the pelvic and abdominal cavities.

**Figure 4:**
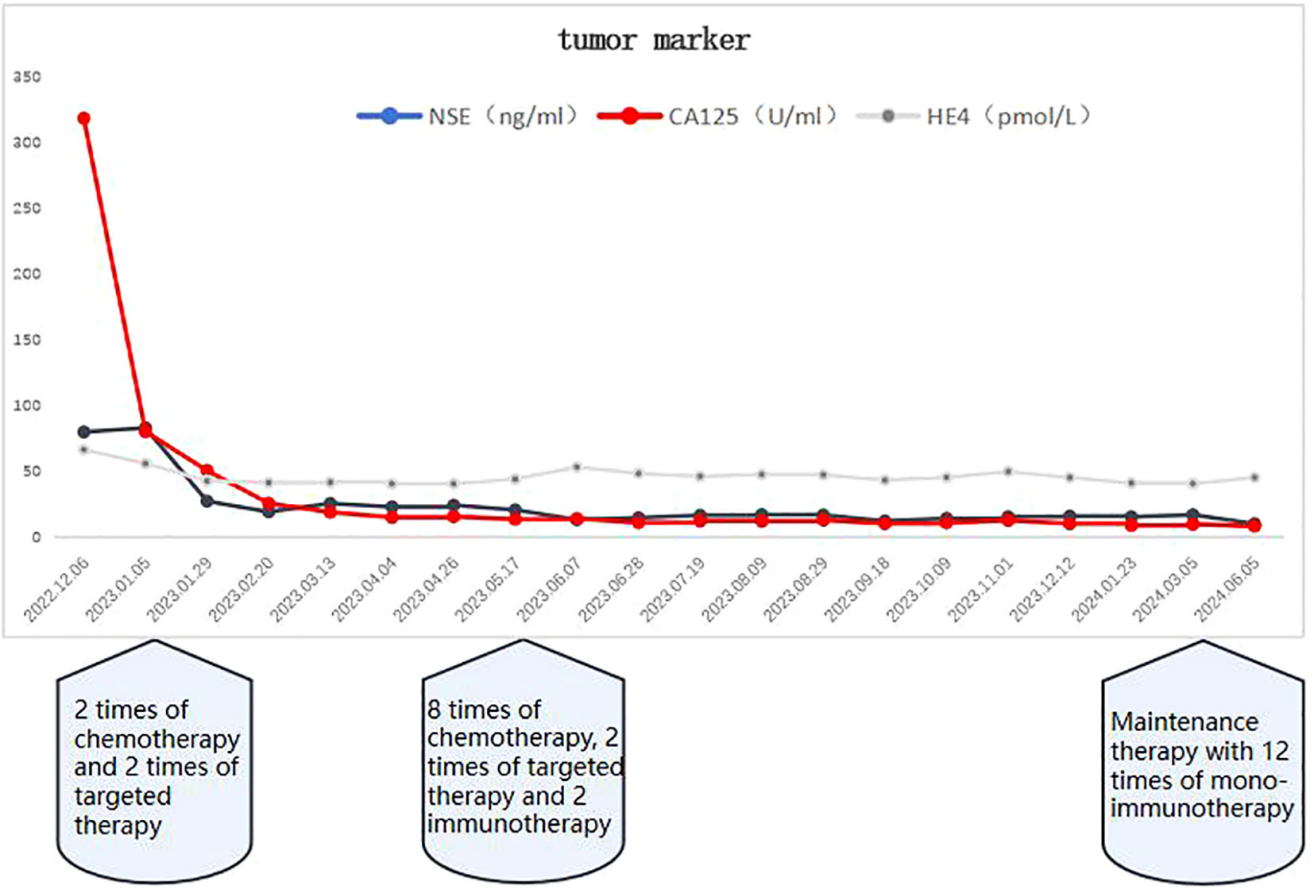
As of June 2024, the tumor markers CA125 and NSE had returned to normal after discontinuation of tiralizumab monotherapy maintenance therapy.

Patient female, 48 years old, married and childless, history of schizophrenia for 20 years, currently on oral olanzapine treatment, In 2018, she underwent laparoscopic debulking of right ovarian cyst in our hospital due to chocolate cyst of ovary. She was admitted to our department in December 2022 due to “sudden abdominal distension for 2 days, abnormal vaginal bleeding for 1 day, and discovery of pelvic mass for 6 hours”. Gynecological ultrasound showed: hypoechoic uterus in the upper right side of the uterus, about 83*65*55mm in size, with irregular morphology, and hypoechoic pelvis in the left side of the pelvic cavity, about 80*43*46mm in size, with unclear boundaries. Computed tomography (CT) scan of the whole abdomen: multiple occupations in the right subperitoneum and pelvis, the large one was about 71*56mm, and there was a large amount of fluid in the abdominal cavity and pelvis (**Figure 1A**). Further, he performed puncture drainage of the abdominal cavity and puncture biopsy of the pelvic mass, and the paraffin-embedded pathology of ascites sediment suggested that more lymphocyte-like cells and a small number of mesothelial cells could be seen, which was suspicious for lymphoma. Pelvic mass puncture biopsy suggested: low differentiated neuroendocrine carcinoma (small cell carcinoma) possible. Immunohistochemical results: tumor cells AE1/AE3 (sporadic +), CAM5.2 (sporadic +), CgA (sporadic +), Syn (weak +), NSE (a few +), CD56 (a few +), PAX-8 (a few +), p53 (80% +), WT1 (Golgi +), Ki -67 (90% +) (**Figure 2**). Gynecological examination: vulva was normal, vagina was smooth, uterus was usually large, no pressure pain, pelvis could be touched on the right side of the mass, the size of about 8*7*6cm, the border was not clear, poor mobility. Triple diagnosis: left posterior uterus can reach the lower pole of the mass, about 7.0*6.0*5.0cm, hard, poor mobility. Adjunctive examination: tumor markers: NSE (neuron-specific enolase) 80.11 ng/ml and CA125 (glycan antigen 125) 318.60 U/ml were significantly higher than the reference value, HE4 (epithelial secretion protein) did not show any abnormality. Lung CT suggested: cords and stripes in the lower lobes of both lungs; a small amount of pleural effusion on the right side, mild thickening of the pleura bilaterally; a small amount of pericardial effusion; calcium ions: 2.04 mmol/L, which was not elevated; gastroenteroscopy did not show any obvious abnormality. PET-CT conclusions: abnormal elevation of glucose metabolism of the right anterior and left pelvic cavity (periungerocele), and the multiple pelvic cavity (left posterocele to presacral) masses, considered malignant; the multiple lymph nodes in the diaphragm on the bilateral sides, the right side of the wall peritoneum, intrahepatic right parietal effusion, splenic peritoneum, multiple masses on the right side of the pelvis (anterior to the psoas major muscle), increased flocculent density of the greater omentum and lesser omentum sheets, and increased glycaemic metabolism of the right hemicolon mesentery were considered to be metastatic. A large amount of fluid in the abdominopelvic cavity with increased glucose metabolism was considered, and the presence of tumor cells in the fluid was considered. (**Figure 3**). According to FIGO (Federation International of Gynecology and Obstetrics) (2014), the diagnosis: stage IVB small cell neuroendocrine carcinoma of the ovary, a multidisciplinary MDT consultation was performed, and it was suggested that either surgery or chemotherapy could be considered; the patient refused to undergo surgery due to personal reasons, and was adamant in requesting chemotherapy. Since December 2022, 8 times of paclitaxel+carboplatin regimen chemotherapy (d1 paclitaxel 270mg IV, d1 carboplatin 550mg IV) were conducted, of which the first 2 times were combined with targeted anti-angiogenic drugs (d3 bevacizumab 300mg IV), and the last 2 times were combined with immunosuppressant drugs (d2 tirilizumab 200mg IV) with an interval of 21 days each time. On the 3rd times of chemotherapy, the patient stopped bevacizumab for economic reasons. Repeat CT of the whole abdomen at the end of chemotherapy showed that the lesion was significantly smaller than before. Maintenance therapy with tirilizumab monotherapy once every 3 weeks was started in June 2023 for a total of 12 times (d1 tirilizumab 200 mg IV). The whole abdomen enhanced CT results showed the lesion continued to shrink and achieved partial remission (PR) (**Figure 1B**). The patient’s symptoms of abdominal distension had completely disappeared, and the pelvic mass was significantly smaller than before, and pelvic and abdominal fluid had disappeared. Tumor markers, CA125 and NSE, were reduced to normal (**Figure 4**), and there were no obvious side effects after chemotherapy, targeted and immunotherapy. Tirilizumab monotherapy maintenance therapy was stopped in June 2024, by which time the patient’s PFS had exceeded 19 months. Subsequent reviews were performed every 3 months, and we will follow this patient closely.

## Discussion

Small cell neuroendocrine carcinoma of the ovary has rapid progression and high malignancy and mortality rates, but due to its rarity, it is mostly reported as a case in the relevant literature. In response to the mechanism of occurrence: it has been reported in the literature that pathogenic mutations in SMARCA4 leading to inactivation of its double allele have been shown to be the driver of almost all SCCOHT cases (>90%) (3, 4). It has also been reported that deletion of SMARCA4 leads to deletion of another SWI/SNF component, SMARCA2. SMARCA4, SMARCA2, and SMARCB1 are members of the mammalian SWI/SNF family of chromatin regulators, and approximately 20% of human malignancies are associated with somatic mutations in the SWI/SNF complex (5). Another literature reported that two somatic mutations were identified in SCCOHT cases using NGS (Next Generation Sequencing): loss of function in the SMARCB1 gene and a missense damage mutation in PTEN, whereas no loss of function in SMARCA4 was found (6). This finding supports the hypothesis that SMARCB1 inactivation can also promote the development of SCCOHT (7). It is hypothesized that the construction of animal models targeting SMARCA4, SMARCA2 and SMARCB1 mutations, and consequently the development of corresponding targeted drugs, would be decisive for the treatment of this disease. Regrettably, due to personal financial reasons, this patient did not undergo tumor gene testing.

Small cell neuroendocrine carcinoma of the ovary is a highly aggressive malignant tumor; small cell neuroendocrine carcinoma of the ovary of the pulmonary type, which is most common in perimenopausal women, does not have hypercalcemia, and almost half of the cases are bilateral tumors; small cell neuroendocrine carcinoma of the ovary of the hypercalcemic type, which has a mean age of onset of 23.9 years, and is accompanied by elevated calcium in two thirds of the patients, and is a unilateral tumor in 99% of the cases, with a characteristic follicular-like interstitial spaces. Long-term survival is very rare and most patients die within 2 years of diagnosis (8). Abdominal pain is the most common clinical presentation, followed by palpable mass, abdominal distension, and nausea and vomiting; the primary diameter of the tumor is usually 14.7 ± 4.8 cm, affecting predominantly the right ovary (64.0%).The mean value of the serum level of CA125 tumor marker is 176 U/ml (9). The diagnosis of small cell neuroendocrine carcinomas (SCNECs) depends on the histologic features, the typical pattern being diffuse follicular sheets of small, tightly packed cells with scanty cytoplasm. Large cell variants consisting of mostly cytoplasm-rich cells are rare (8).NE cells are highly specialized neural-like cells, and neuron-specific enolase (NSE), chromogranin A (CgA), synaptophysin (Syn), and CD56 are the more sensitive markers of neuroendocrine tumors, and IHC staining for NE markers is locally positive or even negative (10). In our case, the patient presented with marked abdominal distension, multiple foci in the pelvic and abdominal cavities with distant metastases, accompanied by a large amount of pelvic and abdominal fluid, CA125 was significantly higher than normal, and NSE, CgA, Syn, and CD56 were all positive. However, because the patient had a pelvic mass puncture biopsy, the tissue sampling was limited, most of the tissue was tumor necrosis, the cell differentiation was very poor, and the unique follicle-like structure had not yet been seen, combined with the immunohistochemistry results, the patient was diagnosed as ovarian small cell neuroendocrine carcinoma. The patient was a perimenopausal woman with a small amount of pleural effusion on the right side, mild thickening of bilateral pleura, blood calcium was not elevated, and the tumor was bilateral, which was considered to be small cell neuroendocrine carcinoma of the ovarian lung type.

Small cell neuroendocrine carcinoma of the ovary has a recurrence rate of up to 65.1%, usually occurring within 11.5 ± 13.3 months of initial treatment, and mainly affecting the abdomen and lower pelvis. Treatment for recurrence includes surgery, chemotherapy and radiotherapy (9). Usually, patients with age >30 years, normal preoperative serum calcium, tumor size< 10 cm, and lack of large cells in pathology have a somewhat better prognosis. In contrast, the later the FIGO stage and the higher the metastasis rate, the poorer the prognosis usually is (8). In a retrospective analysis of 47 patients with SCCOHT at MD Anderson Cancer Center, the median OS was found to be 14.9 months. the earlier the FIGO stage, the better the prognosis (median OS: 35.3 months for stage I patients compared to only 3.3 months for stage IV patients) (11). It has been documented that hypercalcemia associated with SCCOHT subsides after complete surgical resection, and if it is elevated again it is often indicative of disease recurrence, and therefore can be used as a potential tumor marker for early surveillance of recurrence in such cases (12). CA125, although a major tumor marker for ovarian cancer, does not show any specific manifestations in neuroendocrine carcinoma of the ovary.

As small cell neuroendocrine carcinoma of the ovary is clinically rare, most of them are reported as individual cases or small samples, and there is a lack of studies with large sample data. The prognosis is poor and short-term mortality after diagnosis is high. Maria et al. (6) in 2022 reported a case of a young woman with somatic SMARCB1 mutation in SCCOHT who died 11 months after the first symptom onset despite high dose chemotherapy after stem cell transplantation.

In terms of treatment, there is no effective treatment for small cell NE cancer of the ovary. Multimodal combination therapy including initial tumor cytoreduction, radiotherapy and chemotherapy is still mostly used. The choice of regimen is extrapolated from data on small cell lung cancer, and the most commonly used adjuvant chemotherapy is based on the combination of cisplatin and etoposide (13). Paclitaxel combined with cisplatin regimen chemotherapy is usually applied for the treatment of advanced tumors (14). It has also been reported in the literature that undergoing stem cell transplantation (SCT) in combination with tumor cytoreduction combined with postoperative chemotherapy with the addition of high-dose chemotherapy (HDC) significantly improves the overall survival of patients (15). Another prospective trial published in 2007, a multicenter cohort study of 27 patients with SCCOHT treated with a multiagent combination for four to six cycles of therapy followed by tumor cytoreduction, and receiving HDC and autologous SCT in complete remission with a 3-year overall survival of 49% (16), also demonstrated the importance of multimodality combination therapy. In addition, radiation therapy is rarely used in such patients, but some studies have shown that it improves survival (17).

Bevacizumab targets vascular endothelial growth factor-A (vascular endothelial growth factor-A (VEGF-A) monoclonal antibody, which has been approved for the treatment of ovarian cancer in several countries. The addition of bevacizumab to first-line chemotherapy for ovarian cancer, mainly for patients with FIGO stage II-IV, and the continuation of maintenance therapy with bevacizumab after completion of chemotherapy can extend the median progression-free survival (PFS) of advanced patients by 2-4 months (18, 19). Tirelizumab, an anti-PD-1 monoclonal antibody, exerts promising efficacy in the treatment of adult advanced solid tumors with microsatellite highly unstable (MSI-H) or mismatch repair gene-deficient (dMMR) phenotype. In our report, according to the FIGO stage IVB, the patient refused surgery due to personal reasons, and was treated with 8 cycles of paclitaxel + carboplatin chemotherapy, combined with targeted and immunotherapy, and the tumor markers of NSE and CA125 were within the normal range, and the pelvic and abdominal cavity enhanced CT mass and metastatic foci were significantly reduced compared with the previous ones, and ascites almost completely disappeared. and immunotherapy can be hypothesized that chemotherapy combined with targeted and immunotherapy plays a key role in the patient’s prognosis. After checking the related literature, no case of small cell neuroendocrine carcinoma of ovary applying anti-angiogenic drugs at the same time has been found for the time being, and this patient’s treatment plan of chemotherapy combined with targeted and immunotherapy can be regarded as the first of its kind. However, due to the limited number of this type of disease, we need multi-center and large data samples to support our treatment plan. We will also keep a close eye on this patient and continue to follow her prognosis.

In recent years, the introduction of tumor immunotherapy and targeted agents has brought a wide range of prospects for the treatment of this disease. Evidence suggests that the patient’s immune system plays an important role in the pathogenesis and treatment of neuroendocrine cancers, as immune cells can infiltrate the tumor microenvironment and lead to tumor-associated inflammation. Immunotherapy is the most effective method to treat the inflammatory tumor microenvironment (20). Tumors with microsatellite instability and high tumor mutational load have been reported to exhibit more mutations and neoantigens that promote tumor-infiltrating lymphocyte aggregation, which in turn leads to programmed cell death protein 1 (PD-1)/programmed cell death-ligand 1 (PD-L1) overexpression (21). All gynecologic SCNEC have similar clinical behaviors and histopathological manifestations and closely resemble small cell lung cancer (SCLC), although they have different etiologies and risk factors. Based on SCLC management and retrospective studies, the combination of immune checkpoint inhibitors (ICIs) and cytotoxic chemotherapy has theoretically become a therapeutic strategy of exploratory significance. Yuan-Xue Zhu et al (22) reported a case of combination of the immune checkpoint inhibitor, tirilizumab, with etoposide and cisplatin chemotherapy (EP) for the treatment of small-cell ovarian NE carcinoma in 2022 and achieved favorable efficacy. Another case reported that for small cell neuroendocrine carcinoma (SCNEC) of the ovary, the combination of chemotherapy and immunotherapy on the basis of surgery resulted in progression-free survival up to 27 months (23). It can be hypothesized that the combination of chemotherapy and immunotherapy may play a positive role in the treatment of small cell neuroendocrine carcinoma of the ovary. Because of its limited number of cases, whether immunotherapy combined with chemotherapy can bring clinical benefits to patients deserves further multicenter and multiregional clinical trials.

## Conclusion

Ovarian neuroendocrine carcinoma is extremely rare in clinical work, with rapid progression and extremely poor prognosis, and the final diagnosis is usually made by lesion puncture or postoperative pathology combined with immunohistochemistry results (e.g., NSE-positive, CgA-positive, Syn-positive, etc.). Due to the rarity of cases, there is no standardized diagnosis and treatment protocol. Surgery, chemotherapy combined with immune and targeted, radiotherapy, stem cell transplantation, high-dose multi-drug chemotherapy and other comprehensive treatments are usually used. Combined with our case, although surgery was not performed for personal reasons, chemotherapy combined with targeted and immunotherapy achieved significant efficacy and is worth further promotion. The postoperative survival rate is correlated with the patient’s age, clinical stage, treatment method and histologic type and lesion size, all of which are correlated. Combined with previous literature, blood calcium can be used as an indicator to monitor recurrence after SCCOHT. In addition, the use of genetic testing to find mutated genes and the development of new targeted drugs will play an important role in promoting the treatment of this disease.

## Data Availability

The original contributions presented in the study are included in the article/supplementary material. Further inquiries can be directed to the corresponding author.
